# Survival from cancer of the uterine cervix in England and Wales up to 2001

**DOI:** 10.1038/sj.bjc.6604590

**Published:** 2008-09-23

**Authors:** H C Kitchener

**Affiliations:** 1 School of Cancer and Imaging Sciences, University of Manchester, Research Floor, St Mary's Hospital, Whitworth Park, Manchester M13 0JH, UK

## Clinical presentation

Cervical cancer is an increasingly rare cancer in the United Kingdom because of the success of the National Screening Programme. It is much less likely to occur in previously screened women than unscreened women and although it most commonly presents with clinical symptoms, it may be identified during the investigation of a screening cytology abnormality, that is, by colposcopy. When it presents clinically, this usually involves abnormal patterns of vaginal bleeding in premenopausal women or postmenopausal bleeding in older women. There may also be an unpleasant vaginal discharge. When the cancer is more advanced it may present with low abdominal or buttock pain due to pressure on the lumbosacral nerve plexus.

## Diagnosis and treatment

Carcinoma of the cervix is usually clinically obvious on gynaecological examination and a small biopsy may be sufficient to confirm the diagnosis and determine the histotype. On the occasions when it is screen detected, that is, only detected because the affected woman had a routine cervical smear test, it may not be clinically apparent and may require a colposcopic examination. Under these circumstances the tumour may be very early in its natural history, at the microinvasive stage, and requires a so-called ‘cone biopsy’ to assess both depth of invasion and lesion width.

There are two main elements in the diagnostic process, which enable optimal treatment planning:

1. To stage the disease using the FIGO staging system, by evaluating its extent by means of thorough clinical examination, chest X-ray and possibly cystoscopic examination. Magnetic resonance scanning is a valuable supplement to clinical staging particularly in determining the local extent of tumour and identifying obvious node enlargement.
2. To determine the histotype for example, squamous or adenocarcinoma, the degree of differentiation and the presence of lymph vascular channel involvement.

During the past 20 years, three key developments have taken place in cervical cancer management. The most significant therapeutic advance has been the advent of chemoradiation, in which a relatively small dose of platinum chemotherapy is given weekly, concurrently, with external beam radiation. A number of randomised trials published in 1999 confirmed a significant survival benefit for chemoradiation and this has been the recommended standard of care since 2000 in the United Kingdom. The second development has been fertility sparing surgery, which is most appropriate for women who wish to retain fertility and who have tumours less than 2 cm across. In such cases the uterine corpus can be retained and a successful pregnancy may result. The third development, and in some way the most important, has been the determination and national guidance that the management of cervical cancer should be undertaken by specialised multidisciplinary teams. This has improved access to accurate pathological diagnosis, expert treatment planning, and specialised surgical and non-surgical oncological care (NHS Executive, 1999). There was evidence in the past that access to expert care was unequal with unequal outcomes (Wolfe *et al*, 1996) but with the full implementation of Improving Outcomes Guidance, this should be resolved.

## Interpretation of survival patterns

Improvements in survival may reflect either a shift in stage towards diagnosis earlier in the natural history of cancer, therapeutic advances, or improvement in access to best care. In cervical cancer all three apply, with more screen-detected early disease, improved treatment of advanced disease with chemoradiation and widespread access to expert teams in gynaecological cancer centres.

One-year survival is probably determined by the proportion of women who present either with very aggressive disease, which is unresponsive to treatment, or such advanced disease that optimal therapy is not possible. Five-year survival is more a measure of (a) stage at diagnosis that determines the proportion of curable tumours and (b) quality of therapy, which achieves longer lasting control. In general, if cancer of the cervix relapses, this generally occurs within 3 years and recurrence thereafter is unusual. The treatment of recurrence is usually surgical salvage or irradiation in previously non-irradiated patients but either way the results are poor, overall.

The improvement in 1- and 5-year survival rates during the 1970s and 1980s could well be due to improved access to expert radiotherapy and to improved healthcare generally, which means fewer untreatable women at presentation. The effect of a highly aggressive subset of tumours has probably not changed significantly over the past 40 years. The improvement in survival in the women diagnosed between 1986 and 1990 possibly reflects a larger proportion being screen detected, that is, some early prevalent disease being picked up at a more curable stage by the outset in 1988 of the National Cervical Screening Programme. This effect will have been sustained during the 1990s. The lack of any increase in survival since then is probably a reflection of the stable 20% or so of the female population who decline to take up cervical screening. Any significant and widely implemented advances in treatment up to the late 1990s, may result in modest improvements in survival; for example, as a result of national adoption of chemoradiation. Only time will tell but the projected survival data do not apparently point to this.

## Deprivation gap

Cancer of the cervix remains a more prevalent disease among socially deprived women. This is probably due to a combination of riskier lifestyle behaviour such as cigarette smoking, earlier onset of sexual intercourse and most importantly, poorer uptake of cervical screening. Other influences during the last 10 years, which may be associated with social deprivation are HIV-positive women and unscreened immigrant populations. There is little evidence that any of these risk factors, which are more prevalent among socially deprived women, have changed suggesting a low likelihood of a reduction in incidence rates. Survival will be influenced by screening, as screen detected cancers are generally at an earlier stage, and up until 2000 access to optimal cancer care was uneven across the country.

## Closing the deprivation gap

### Incidence

Greater awareness of sexual health, reduced smoking habit and education regarding cervical screening are all key to reducing the risk of acquiring cervical cancer.

Vaccination against HPV, however, has the greatest potential to reduce deaths from cervical cancer by preventing genital infection by the high-risk oncotypes of HPV, particularly 16 and 18. It is generally reckoned that worldwide, these HPV strains are responsible for approximately 70% of cervical cancer. If these infections were prevented then the precursor lesions of cervical cancer and the cancer itself should be prevented. Recent publications from two major global trials of prophylactic vaccines against HPV 16 and 18 indicate that HPV vaccines are highly effective at preventing type specific infection and type specific cervical intraepithelial neoplasia. The data from these trials have resulted in a commitment from the UK Government, among others, to provide funding for a national vaccination programme of 12- to 13-year-old females, with a one-off catch-up to the age of 18 years over a 3-year period. There are a number of unknowns at the moment, which require further work. These include longevity of vaccine protection, cross coverage of the other HPV types and whether prior HPV exposed women, not currently infected with HPV 16 or 18, can be protected by preventing re-infection. Any impact this has on cervical cancer death rates would take years to be felt but vaccinating older females on the threshold of cervical screening could have a more rapid impact in terms of reduced incidence of CIN. Because HPV 16 and 18 are only responsible for 60% of CIN3, cervical screening will need to continue and with new protocols considered.

### Survival

The best opportunity to influence survival will be a trend towards earlier presentation, brought about by better screening, resulting in more screen-detected cases and with fewer advanced cases presenting only after the establishment of abnormal bleeding and pain. Screen detected cancers are relatively less common in deprived areas of the West Midlands (G Lawrence, personal communication) and this will have an impact on survival. Universal access for all women to receive care in a specialist centre for cervical cancers as a result of the Cancer Plan, means that optimal care should be provided to socially deprived women, improving disease outcomes. Nevertheless, screening offers the strongest strategy to close the deprivation gap in survival.

### Overall comment

Death rates from cervical cancer have fallen dramatically over the past 20 years and for those unfortunate enough to acquire the disease, survival has improved. The deprivation gap in incidence would reduce if more women avoided tobacco and attended screening. With respect to survival, more screen detection of early cancer, through greater uptake of screening, access to optimal therapy and centralised expert multidisciplinary care, combine to offer the opportunity to close the deprivation gap.

A national audit of cervical cancers is urgently required to map the disease in terms of stage, and to identify why the means to prevent this preventable disease have failed. Ultimately, cervical cancer could be virtually eradicated though prophylactic vaccination of young adolescents combined with appropriate cervical screening from the age 25. Implementing a successful vaccination policy will be challenging but it may be the best hope for protecting all women.
